# Supplementary material for: Rational design of a flavoenzyme for aerobic nicotine catabolism
Source: mBio. 2024 Aug 27;15(10):e02050-24. doi: 10.1128/mbio.02050-24 (PMC11481913; doi:10.1128/mbio.02050-24)
Supplement: Supplemental material — Fig. S1 to S9; Tables S1 to S6. [file mbio.02050-24-s0002.docx]

Supplementary Materials for

**Rational Design of a Flavoenzyme for Aerobic Nicotine Catabolism**

Haiyang Hu^1^**^†^,** Zhaoyong Xu^1^**^†^**, Zhiyao Zhang^2^, Peizhi Song^1^, Frederick Stull^2^, Ping Xu^1^, and Hongzhi Tang^1^*

^1^State Key Laboratory of Microbial Metabolism, Joint International Research Laboratory of Metabolic and Developmental Sciences, and School of Life Sciences and Biotechnology, Shanghai Jiao Tong University, Shanghai, People's Republic of China

^2^Department of Chemistry, Western Michigan University, Kalamazoo, MI, USA

^†^These authors contributed equally to this study

*Corresponding author: H. Tang

Mailing address: School of Life Sciences & Biotechnology, Shanghai Jiao Tong University, Shanghai 200240, P. R. China

**Email:** [tanghongzhi@sjtu.edu.cn](mailto:tanghongzhi@sjtu.edu.cn); Tel: +86-21-34204066; Fax: +86-21-34206723

**This PDF file includes:**

Supplementary Figs. S1 to S9

Supplementary Tables S1 to S6


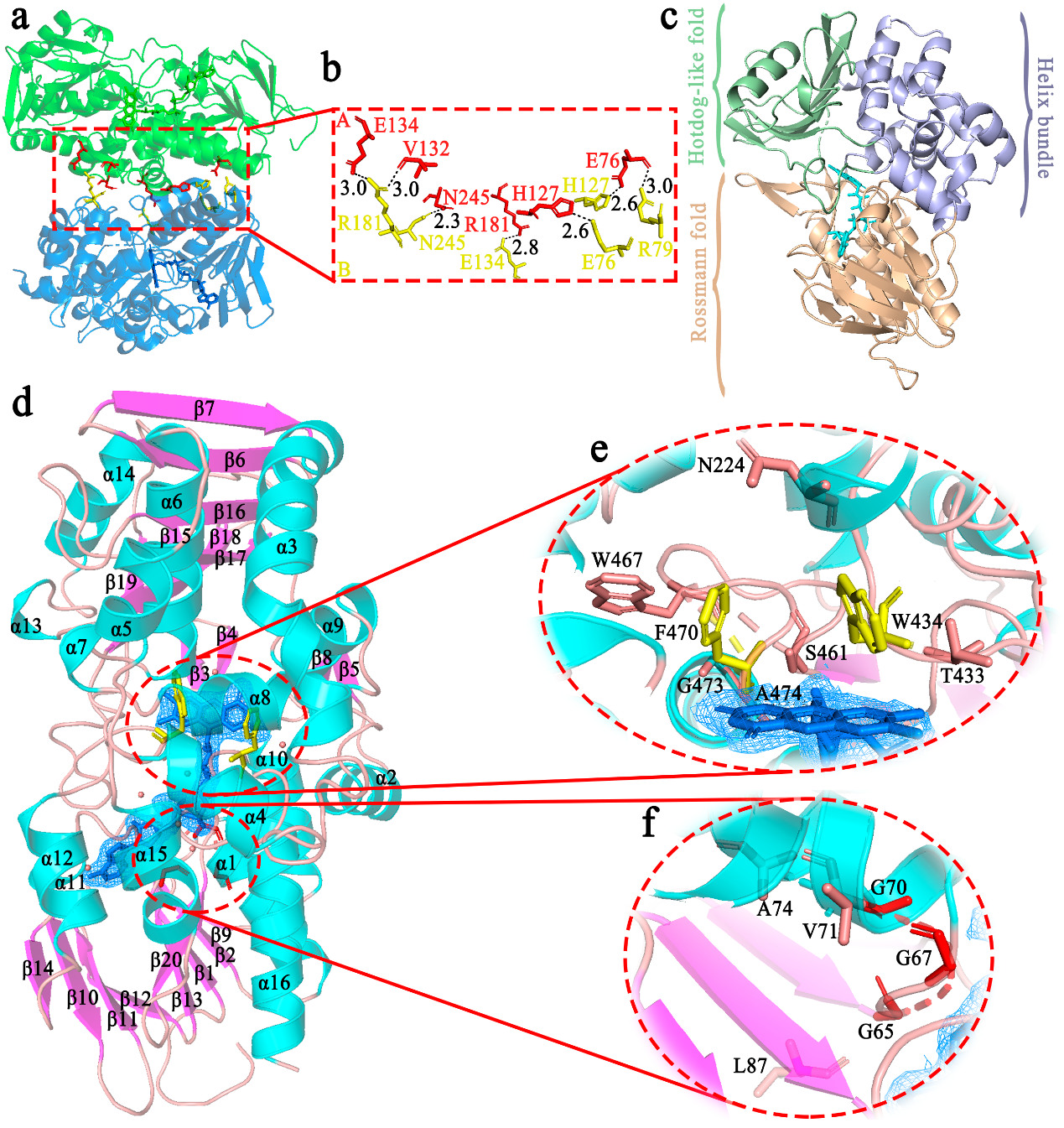


**Supplementary Fig. 1 Overall structure of Pnao.** **(a)** Pnao was a dimer in solution and the amino acids at the interface are shown in red and yellow of these 2 monomers and details can be seen in **(b)**. **(c)** There are 3 individual domains to Pnao, the Rossmann fold in wheat is related to FAD (in cyan) binding, the hotdog-like fold in pale green, and the helix bundle in light blue are related to substrate binding. **(d)** The 3D structure of Pnao. α-helixes are in cyan, β-strands are in magenta, loops are in salmon and FAD is in marine, with the *2F_O_-F_C_* electron density map contoured at 1σ. **(e)** Trp434 and Phe470 colored in yellow flanking the *re*-side of the flavin isoalloxazine ring constructed a sandwich structure for substrate binding. **(f)** The conserved GXGXXG motif in the FAD superfamily comprised G65, G67, and G70 colored in red.


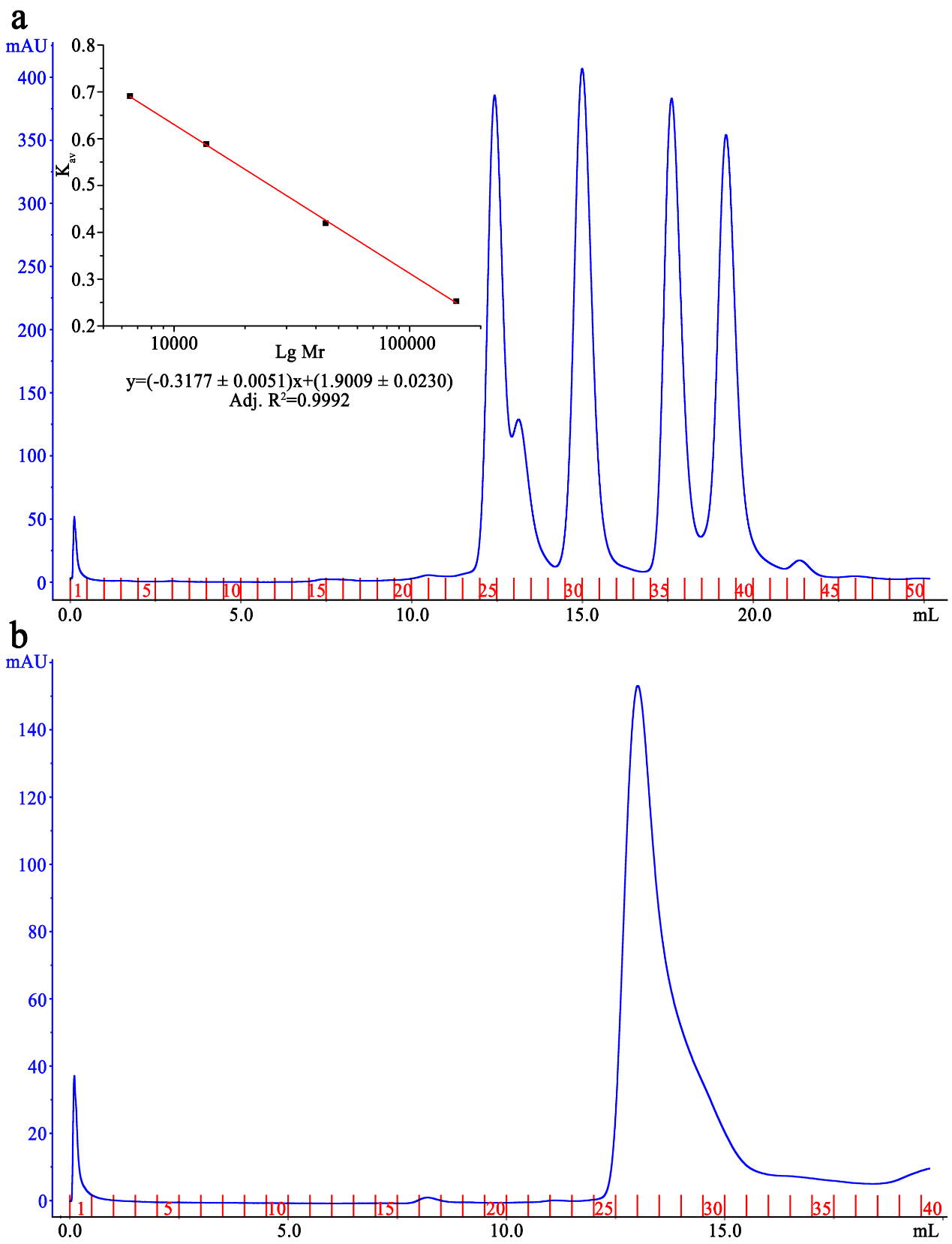


**Supplementary Fig. 2 Size-exclusion chromatograph (SEC) of Pnao.** **(a)** The standard curve of elution out through SEC established the equation between the molecular weight (x) and the K_av_ (y). **(b)** The elution of Pnao.


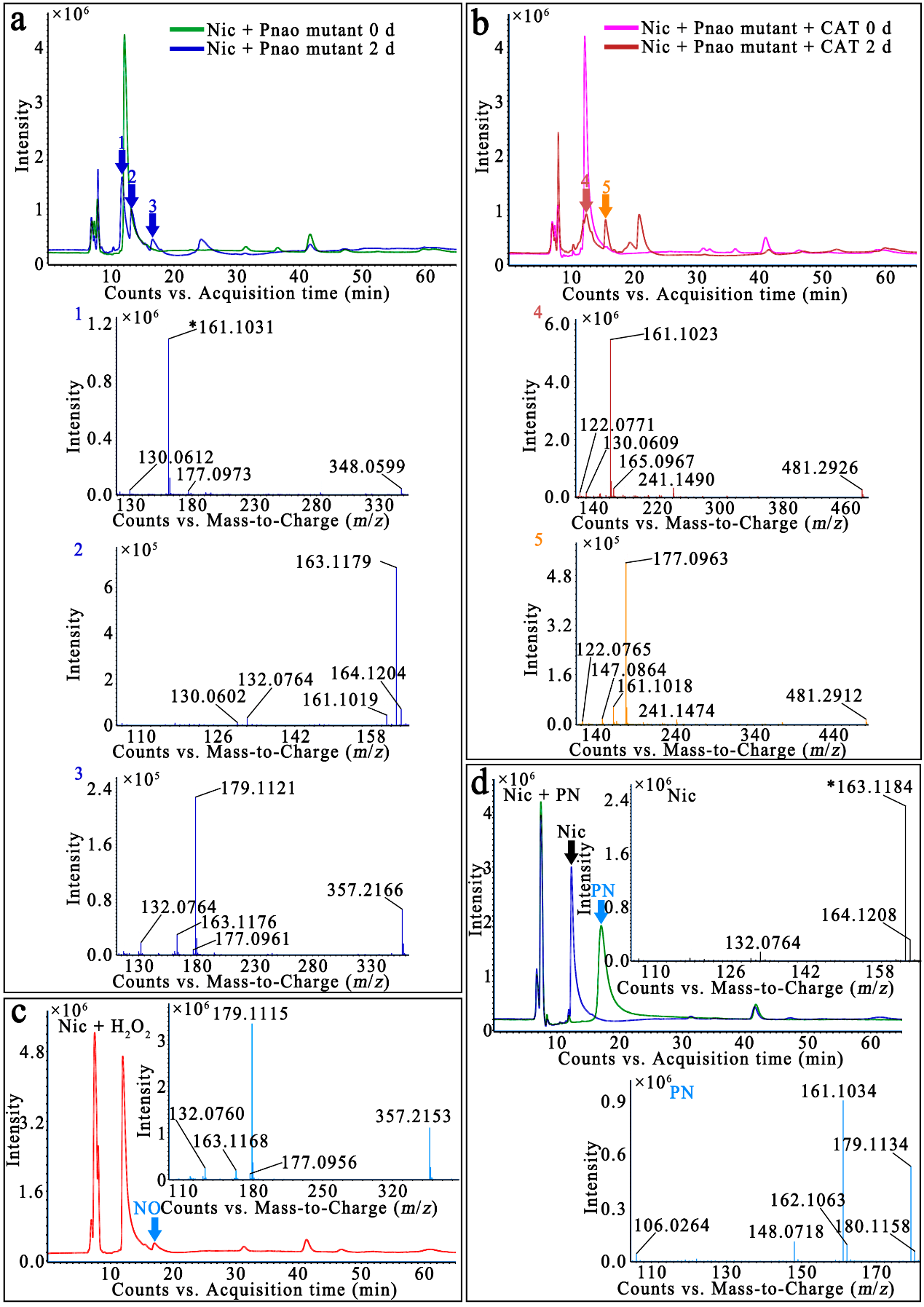


**Supplementary Fig. 3 Liquid chromatography quadrupole time of flight mass spectrum (LC/QToF MS) comparison of nicotine product catabolized by Pnao mutants.** **(a,b)** Total ion chromatographs and mass spectrums of nicotine (Nic) products catabolized by Pnao mutant **(a)** and Pnao mutant added with catalase (CAT, **b**). **(c)** Total ion chromatograph and ion fragments of nicotine-1'-*N*-oxide (NO), product of nicotine and H_2_O_2_. **(d)** Total ion chromatograph and ion fragments of Nic and pseudooxynicotine (PN).


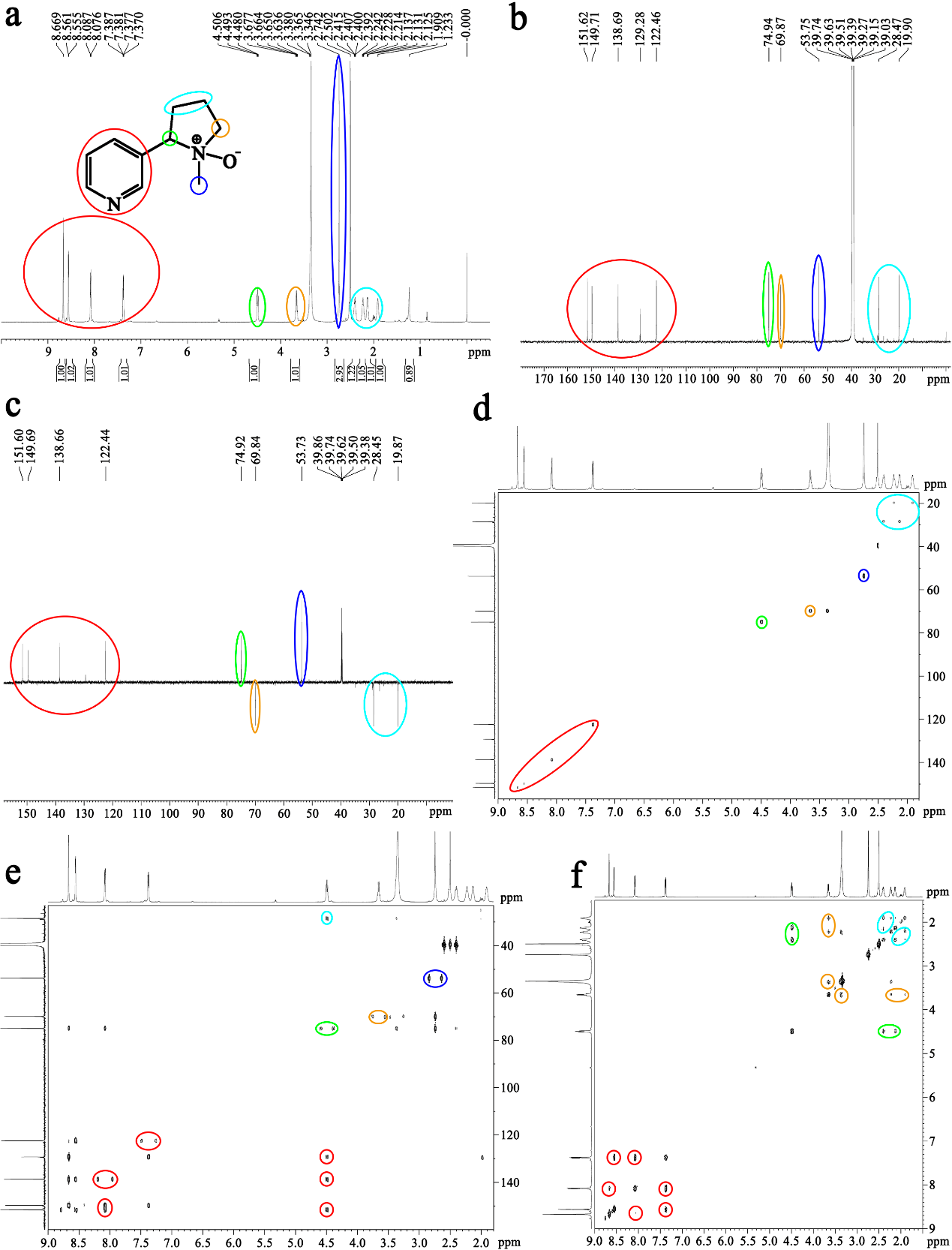


**Supplementary Fig. 4 Nuclear magnetic resonance (NMR) data of product of Pnao mutant catabolizing nicotine.** **(a**–**f)** ^1^H spectrum **(a)**, ^13^C spectrum **(b)**, ^13^C DEPT 135 **(c)**, HSQC **(d)**, HMBC **(e)** and COSY **(f)** of product of Pnao mutant catabolizing nicotine.


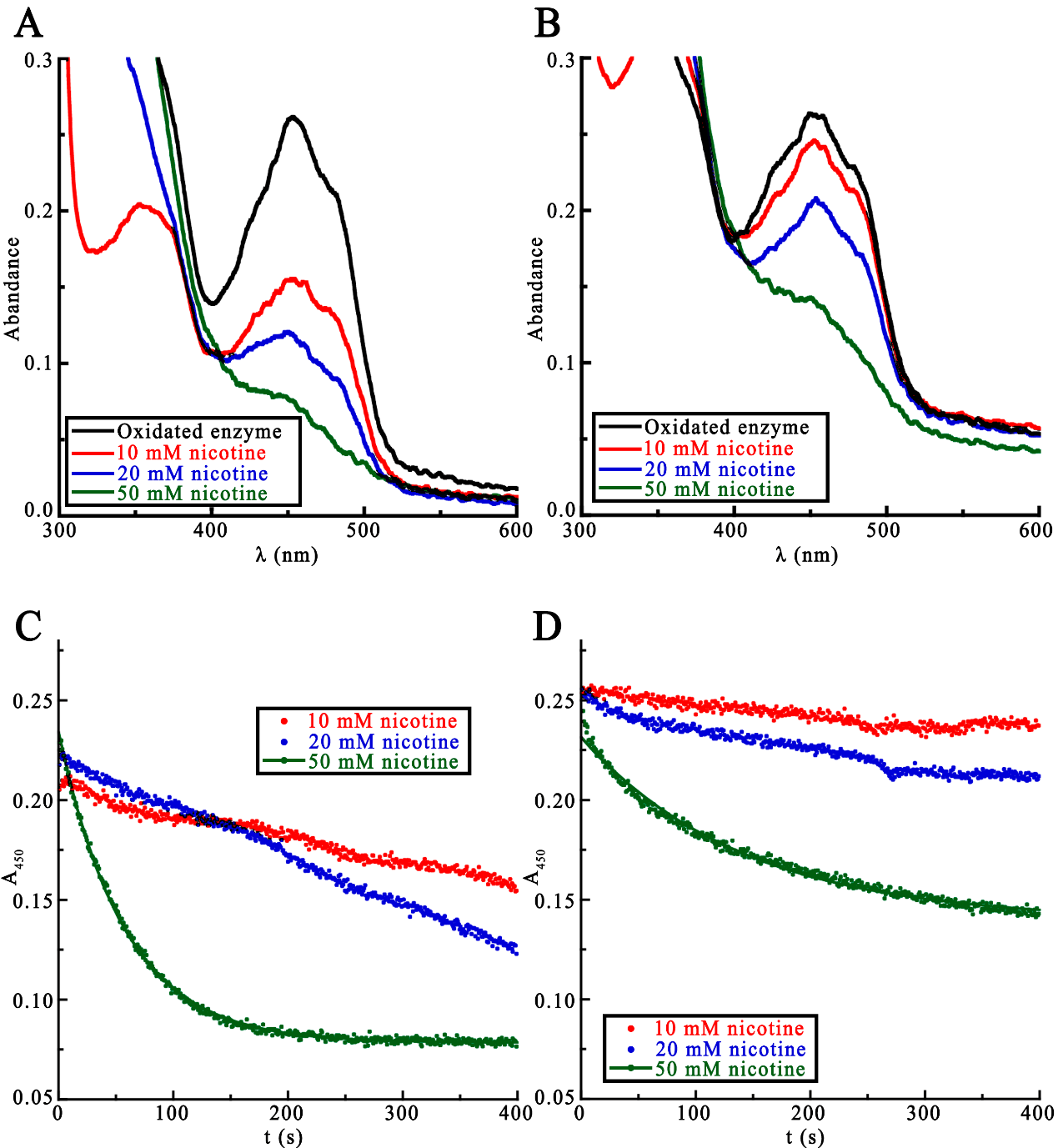


**Supplementary Fig. 5 Stopped-flow data for Pnao variants reacting with nicotine.** **(a,b)** The final absorbance spectrum at 400 seconds upon reacting with various concentrations of nicotine for Pnao-W220Y-N224F **(a)** and wild type **(b)**, respectively. **(c,d)** Reaction traces at 450 nm for Pnao-W220Y-N224F **(c)** and wild type **(d)**, respectively. The traces at 50 mM nicotine were fit to an exponential function, yielding an observed rate constant of 0.017 s^−1^ for Pnao-W220Y-N224F and 0.007 s^−1^ for wild-type Pnao.


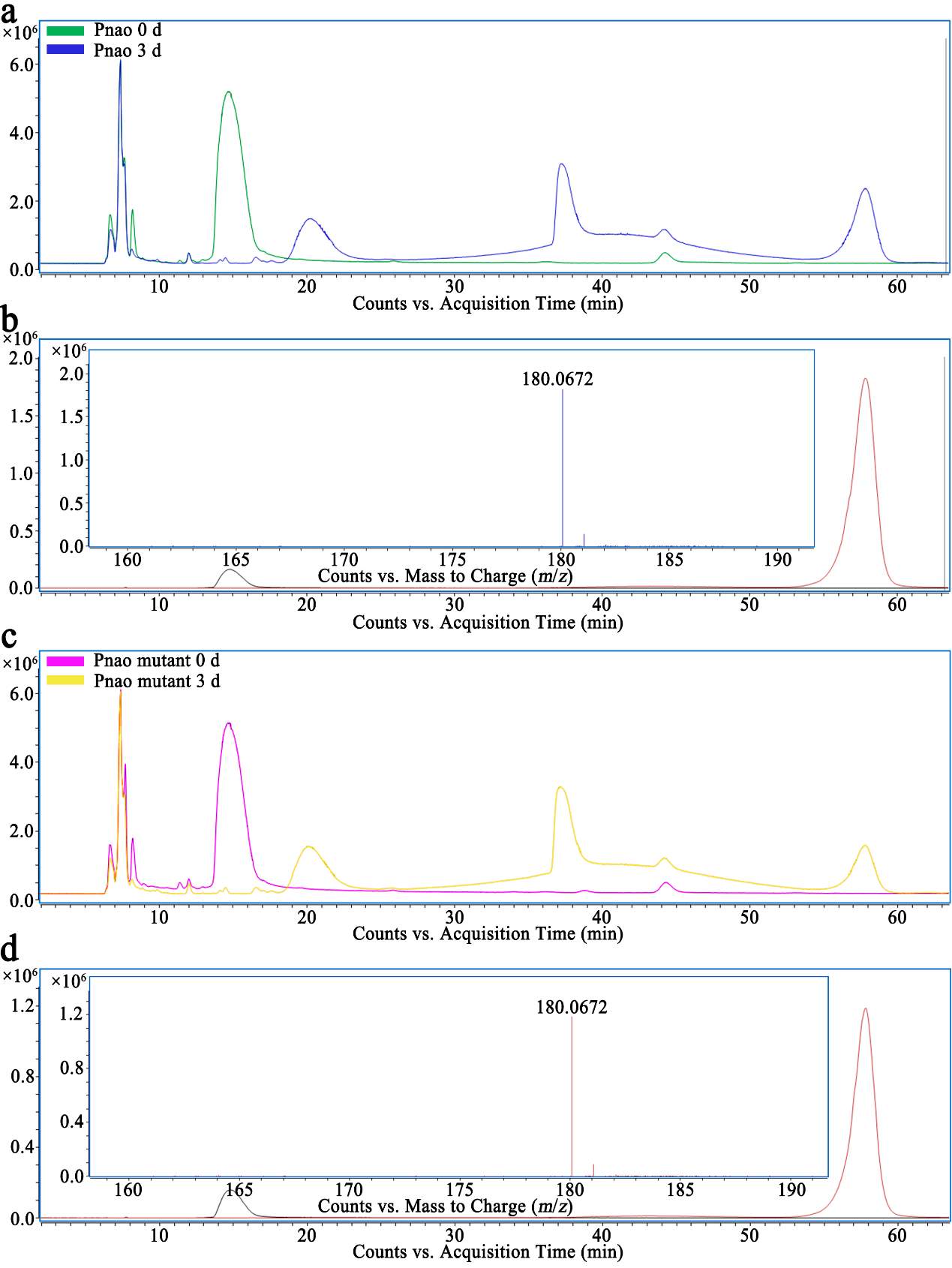


**Supplementary Fig. 6 Liquid chromatography quadrupole time of flight mass spectrum (LC/QToF MS) comparison of PN product catabolize by Pnao wild type or mutants.** **(a,c)** Total ion chromatograph of products of Pnao **(a)** and Pnao mutants **(c)** catabolizing PN. There are some new peaks generated after reaction for 3 d (colored in blue in **a** and yellow in **c**) and the spectrum of them can be matched. **(b,d)** Extract ion chromatograph at *m*/*z* 180.0655 (3-succinoyl pyridine) and related fragments spectrum of Pnao **(b)** and Pnao mutants **(d)**.


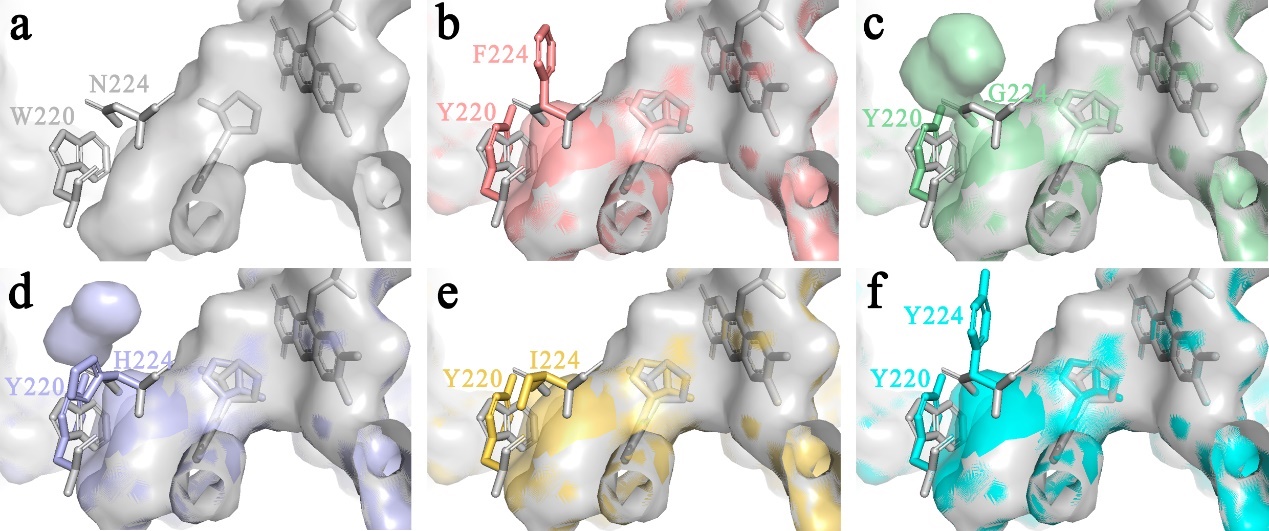


**Supplementary Fig. 7 Wild-type Pnao and computer simulation of the mutant structures.** **(a)** The local structure around Trp220 and Asn224 in Pnao. **(b**–**f)** Superposed structures showed differences in the regions around the mutation sites between Pnao (gray) and Pnao-W220Y-N224F (**b**, salmon), Pnao-W220Y-N224G (**c**, light green), Pnao-W220Y-N224H (**d**, light blue), Pnao-W220Y-N224I (**e**, light yellow), and Pnao-W220Y-N224Y (**f**, cyan). The nicotine molecule is shown in stick form docked into Pnao or the derivative mutants.


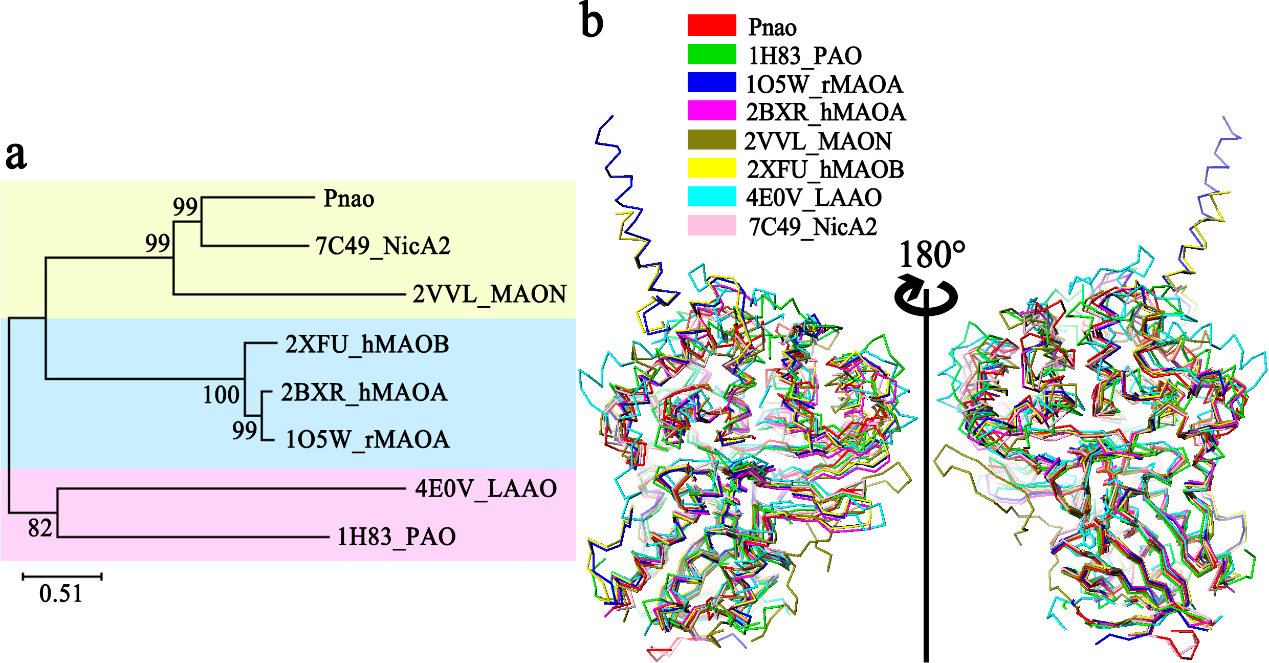


**Supplementary Fig. 8 Phylogenetic tree and structural alignment of Pnao and related enzymes.** **(a)** Phylogenetic tree showing relationships between Pnao, NicA2, monoamine oxidase N (MAON), human monoamine oxidase A (hMAOA), rat monoamine oxidase A (rMAOA), human monoamine oxidase B (hMAOB), l-amino-acid oxidase (LAAO), and polyamine oxidase (PAO). **(b)** Structural alignment of Pnao, NicA2, MAON, hMAOA, rMAOA, hMAOB, LAAO, and PAO. The root mean square deviation (RMSD) values between Pnao and the other enzymes were as follows: 0.988 Å (MAON), 1.143 Å (NicA2), 1.798 Å (hMAOA), 1.815 Å (rMAOA), 2.011 Å (hMAOB), 2.673 Å (PAO), and 2.987 Å (LAAO).


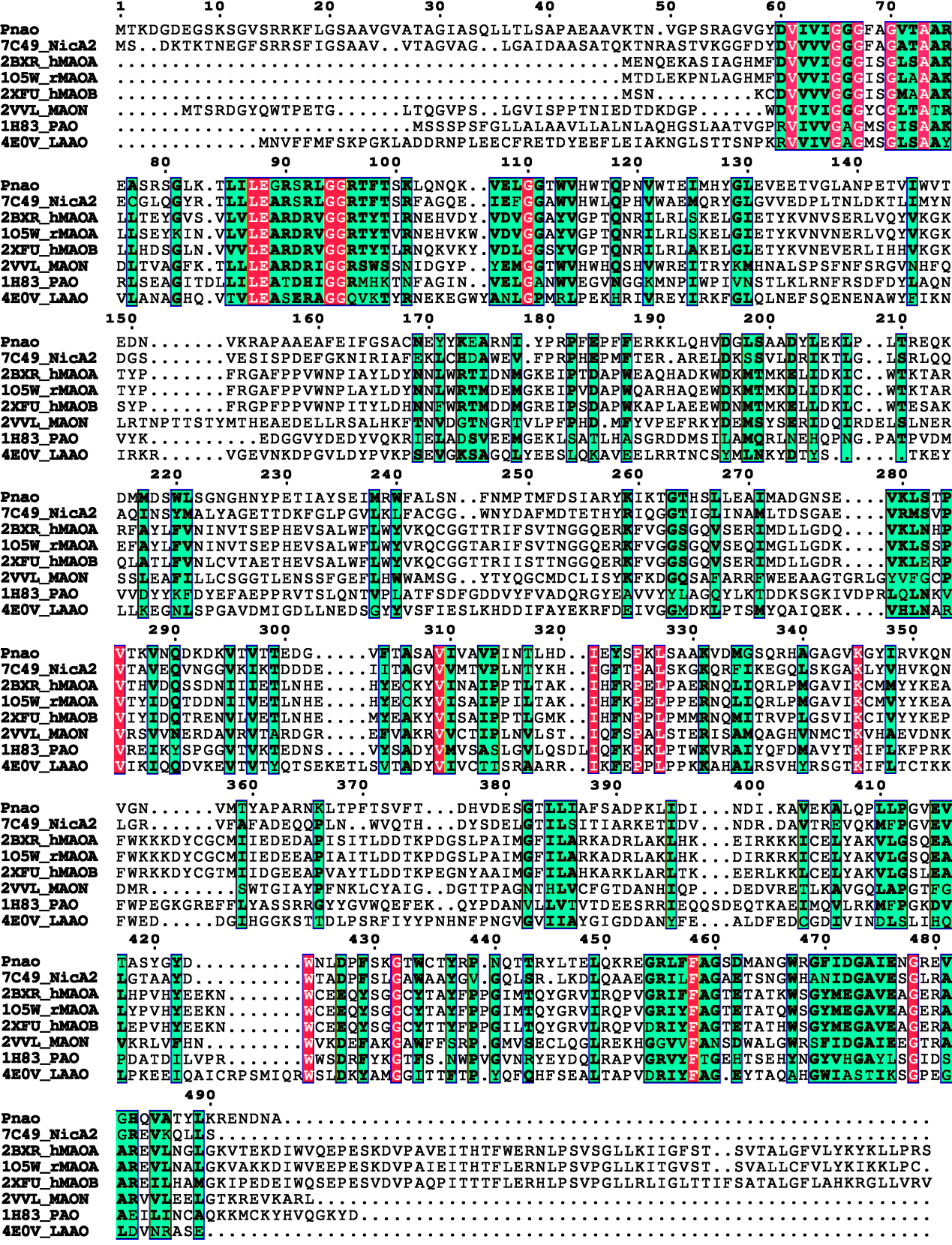


**Supplementary Fig. 9 Amino acid sequence alignment of Pnao and related enzymes.** Based on the phylogenetic tree in Fig. 1b, seven related enzymes with known structures were selected for sequence alignment. Amino acids highlighted in green have high similarity between sequences (bolded residues are preferred) and those highlighted in red are identical between sequences.

**Supplementary Table 1 Amino acid sequence blast of Pnao**

| **Name** | **Score** | **Cover** | **Identity** | **Accession** |
| --- | --- | --- | --- | --- |
| Pseudooxynicotine oxidase | 791.0 | 100% | 78.67% | H8ZPX1.1 |
| Nicotine oxidoreductase | 301.0 | 96% | 39.05% | WP_013973880.1 |
| Monoamine oxidase N | 173.0 | 85% | 27.43% | P46882.1 |
| Monoamine oxidase A | 91.7 | 85% | 23.06% | Q6Q2J0.1 |
| Monoamine oxidase B | 84.7 | 83% | 24.56% | P56560.4 |
| L-amino-acid oxidase | 80.5 | 82% | 25.55% | A8QL51.1 |
| Putrescine oxidase | 55.1 | 82% | 21.32% | P40974.1 |
| Polyamine oxidase B | 53.1 | 84% | 20.78% | A0A0P0XM10.1 |
| Protein FLOWERING LOCUS D | 44.7 | 10% | 40.35% | Q9CAE3.1 |
| Lysine-specific histone demethylase 2 | 43.1 | 45% | 22.89% | Q8CIG3.1 |

**Supplementary Table 2 The FAD cofactor binding ratio of Pnao mutants**

| **Protein** | **Protein concentration (mM)** | **FAD concentration (mM)** | **FAD/ Protein** |
| --- | --- | --- | --- |
| Pnao | 0.087 | 0.054 | 0.62 |
| Pnao-R90A | 0.093 | 0.072 | 0.77 |
| Pnao-R96A | 0.087 | 0.069 | 0.79 |
| Pnao-S461A | 0.088 | 0.054 | 0.61 |
| Pnao-R90A-R96A | ND | ND | ND |
| Pnao-R90A-S461A | 0.091 | 0.071 | 0.78 |
| Pnao-R96A-S461A | 0.069 | 0.051 | 0.74 |
| Pnao-R90A-R96A-S461A | ND | ND | ND |

"ND" means not detected.

**Supplementary Table 3 The enzymatic kinetic parameters of Pnao and its mutants with PN**

| **Protein** | ***K*_m_ (mM)** | ***k*_cat_ (s^-1^)** | ***k_cat_* / *K_m_* (L mol^-1^ s^-1^)** |
| --- | --- | --- | --- |
| Pnao | 0.032 ± 0.001 | 197 ± 1 | 6.1 × 10^6^ |
| Pnao-R96A | 0.056 ± 0.001 | 0.75 ± 0.06 | 1.3 × 10^4^ |
| Pnao-R90A-R96A | ND | ND | ND |
| Pnao-R96A-S461A | 0.083 ± 0.012 | 0.39 ± 0.01 | 4.7 × 10^3^ |
| Pnao-R90A-R96A-S461A | ND | ND | ND |
| Pnao-F388A | 0.054 ± 0.001 | 0.75 ± 0.01 | 1.4 × 10^4^ |
| Pnao-W434A | ND | ND | ND |
| Pnao-W220Y-N224F | 2.6 ± 1.0 | 148 ± 38 | 5.7 × 10^4^ |
| Pnao-W220Y-N224G | 0.15 ± 0.00 | 135 ± 2 | 9.1 × 10^5^ |
| Pnao-W220Y-N224H | 0.45 ± 0.07 | 9.9 ± 1.3 | 2.2 × 10^4^ |
| Pnao-W220Y-N224I | 0.57 ± 0.05 | 383 ± 19 | 6.8 × 10^5^ |
| Pnao-W220Y-N224Y | 0.29 ± 0.01 | 3.1 ± 0.0 | 1.1 × 10^4^ |

"ND" means not detected.

**Supplementary Table 4 Data collection and refinement statistics of Pnao**

| **Parameter** | **Value of Pnao** |
| --- | --- |
| Data collection statistics |  |
| Space group | P 2_1_ 2_1_ 2_1_ |
| Wavelength | 0.97918 |
| Cell dimensions |  |
| a, b, c (Å) | 69.657 89.709 157.583 |
| α, β, γ (°) | 90 90 90 |
| Molecules per asymmetric unit | 2 |
| Resolution range (Å)* | 34.29–2.2 (2.279–2.2) |
| Completeness (%)* | 95.04 (88.17) |
| Redundancy* | 11.9 (9.2) |
| Total no. of observations* | 62,256 (4,741) |
| No. of unique reflections* | 48,413 (4,895) |
| R-merge* | 0.218 (0.653) |
| *I / σI** | 6.94 (1.65) |
| Refinement statistics |  |
| Resolution range (Å) | 34.29–2.2 |
| R-work/ R-free | 0.1655/0.2215 |
| No. of reflections* | 48,412 (4,429) |
| RMSD |  |
| Bond length (Å) | 0.007 |
| Bond angle (°) | 0.90 |
| Ramachandran plot (%) |  |
| Most favored | 95.32 |
| Allowed | 4.68 |
| Disallowed | 0.00 |
| No. of atoms |  |
| Protein | 6774 |
| ligands | 136 |
| Water | 482 |
| Average B-factor |  |
| Protein | 33.11 |
| ligands | 28.56 |
| Water | 36.84 |

*Statistics for the highest-resolution shell are shown in parentheses.

**Supplementary Table 5 Channel priority of Pnao**

| **Tunnel cluster ID** | **Priority** | **Average throughput** |
| --- | --- | --- |
| 1 | 0.379 | 0.379 |
| 2 | 0.331 | 0.331 |

**Supplementary Table 6 Primers**' **sequence used in this research**

| **Primer** | **Sequence** |
| --- | --- |
| Pnao-Δ30-F | AAGAAGGAGATATACCATGGCCATAGCCTCGCAGCTTCTGAC |
| Pnao-Δ30-R | GGTGGTGGTGGTGCTCGAGCGCATTGTCATTTTCTCTTTTTAG |
| Pnao-W220A-F | ATGGATTCCGCGCTCAGTGGAAATGGACATAAT |
| Pnao-W220A-R | TCCACTGAGCGCGGAATCCATCATATCCTTTTG |
| Pnao-N224A-F | CTCAGTGGAGCGGGACATAATTATCCGGAAACT |
| Pnao-N224A-R | ATTATGTCCCGCTCCACTGAGCCAGGAATCCAT |
| Pnao-F388A-F | CTCATTGCAGCGTCAGCCGACCCTAAGTTGATT |
| Pnao-F388A-R | GTCGGCTGACGCTGCAATGAGTAACGTACCGCT |
| Pnao-W434A-F | AAGGGCACTGCGTGCACTTACCGTCCTAACCAG |
| Pnao-W434A-R | GTAAGTGCACGCAGTGCCCTTAGAAAAGGGATC |
| Pnao-F470A-F | TGGCGTGGAGCGATTGATGGAGCAATCGAGAAC |
| Pnao-F470A-R | TCCATCAATCGCTCCACGCCAACCATTGGCCAT |
| Pnao-R90A-F | ATTCTTGAAGGTGCAAGTCGGTTGGGCGGCCGAACT |
| Pnao-R90A-R | GCCCAACCGACTTGCACCTTCAAGAATTAGAGTTTT |
| Pnao-R96A-F | TTGGGCGGCGCAACTTTTACGTCTAAGCTT |
| Pnao-R96A-R | CGTAAAAGTTGCGCCGCCCAACCGACTTCT |
| Pnao-S461A-F | TTTGCAGGCGCAGACATGGCCAATGGTTGG |
| Pnao-S461A-R | GGCCATGTCTGCGCCTGCAAAAAATAGCCG |
| Pnao-R90A-R96A-F | CTTGAAGGTGCAAGTCGGTTGGGCGGCGCAACTTTTACGTCTAAGCTT |
| Pnao-R90A-R96A-R | CGTAAAAGTTGCGCCGCCCAACCGACTTGCACCTTCAAGAATTAGAGT |
| Pnao-W220Y-F | ATGGATTCCTATCTCAGTGGAAATGGACATAAT |
| Pnao-W220Y-R | TCCACTGAGATAGGAATCCATCATATCCTTTTG |
| Pnao-G469A-F | GGTTGGCGTGCGTTCATTGATGGAGCAATCGAG |
| Pnao-G469A-R | ATCAATGAACGCACGCCAACCATTGGCCATGTC |
| Pnao-F470N-F | TGGCGTGGAAATATTGATGGAGCAATCGAGAAC |
| Pnao-F470N-R | TCCATCAATATTTCCACGCCAACCATTGGCCAT |
| Pnao-G469A-F470N-F | GGTTGGCGTGCAAACATTGATGGAGCAATCGAGAAC |
| Pnao-G469A-F470N-R | TCCATCAATGTTTGCACGCCAACCATTGGCCAT |
| Pnao-W220Y-F | ATGGATTCCTATCTCAGTGGAAATGGACATAAT |
| Pnao-W220Y-R | TCCACTGAGATAGGAATCCATCATATCCTTTTG |
| Pnao-G223L-F | TGGCTCAGTCTCAATGGACATAATTATCCGGAA |
| Pnao-G223L-R | ATGTCCATTGAGACTGAGCCAGGAATCCATCAT |
| Pnao-N224Y-F | CTCAGTGGATATGGACATAATTATCCGGAAACT |
| Pnao-N224Y-R | ATTATGTCCATATCCACTGAGCCAGGAATCCAT |
| Pnao-W220Y-G223L-F | ATGGATTCCTATCTCAGTCTCAATGGACATAATTATCCGGAA |
| Pnao-W220Y-G223L-R | ATGTCCATTGAGACTGAGATAGGAATCCATCATATCCTTTTG |
| Pnao-W220Y-N224Y-F | ATGGATTCCTATCTCAGTGGATATGGACATAATTATCCGGAAACT |
| Pnao-W220Y-N224Y-R | ATTATGTCCATATCCACTGAGATAGGAATCCATCATATCCTTTTG |
| Pnao-G223L-N224Y-F | TGGCTCAGTCTCTATGGACATAATTATCCGGAAACT |
| Pnao-G223L-N224Y-R | ATTATGTCCATAGAGACTGAGCCAGGAATCCATCAT |
| Pnao-W220Y-G223L-N224Y-F | ATGGATTCCTATCTCAGTCTATATGGACATAATTATCCGGAAACT |
| Pnao-W220Y-G223L-N224Y-R | ATTATGTCCATATAGACTGAGATAGGAATCCATCATATCCTTTTG |
| Pnao-W220Y-N224F-F | ATGGATTCCTATCTCAGTGGATTTGGACATAATTATCCGGAAACT |
| Pnao-W220Y-N224F-R | ATTATGTCCAAATCCACTGAGATAGGAATCCATCATATCCTTTTG |
| Pnao-W220Y-N224L-F | ATGGATTCCTATCTCAGTGGACTTGGACATAATTATCCGGAAACT |
| Pnao-W220Y-N224L-R | ATTATGTCCAAGTCCACTGAGATAGGAATCCATCATATCCTTTTG |
| Pnao-W220Y-N224I-F | ATGGATTCCTATCTCAGTGGAATTGGACATAATTATCCGGAAACT |
| Pnao-W220Y-N224I-R | ATTATGTCCAATTCCACTGAGATAGGAATCCATCATATCCTTTTG |
| Pnao-W220Y-N224M-F | ATGGATTCCTATCTCAGTGGAATGGGACATAATTATCCGGAAACT |
| Pnao-W220Y-N224M-R | ATTATGTCCCATTCCACTGAGATAGGAATCCATCATATCCTTTTG |
| Pnao-W220Y-N224V-F | ATGGATTCCTATCTCAGTGGAGTTGGACATAATTATCCGGAAACT |
| Pnao-W220Y-N224V-R | ATTATGTCCAACTCCACTGAGATAGGAATCCATCATATCCTTTTG |
| Pnao-W220Y-N224S-F | ATGGATTCCTATCTCAGTGGATCTGGACATAATTATCCGGAAACT |
| Pnao-W220Y-N224S-R | ATTATGTCCAGATCCACTGAGATAGGAATCCATCATATCCTTTTG |
| Pnao-W220Y-N224P-F | ATGGATTCCTATCTCAGTGGACCTGGACATAATTATCCGGAAACT |
| Pnao-W220Y-N224P-R | ATTATGTCCAGGTCCACTGAGATAGGAATCCATCATATCCTTTTG |
| Pnao-W220Y-N224T-F | ATGGATTCCTATCTCAGTGGAACTGGACATAATTATCCGGAAACT |
| Pnao-W220Y-N224T-R | ATTATGTCCAGTTCCACTGAGATAGGAATCCATCATATCCTTTTG |
| Pnao-W220Y-N224H-F | ATGGATTCCTATCTCAGTGGACATGGACATAATTATCCGGAAACT |
| Pnao-W220Y-N224H-R | ATTATGTCCATGTCCACTGAGATAGGAATCCATCATATCCTTTTG |
| Pnao-W220Y-N224Q-F | ATGGATTCCTATCTCAGTGGACAAGGACATAATTATCCGGAAACT |
| Pnao-W220Y-N224Q-R | ATTATGTCCTTGTCCACTGAGATAGGAATCCATCATATCCTTTTG |
| Pnao-W220Y-N224K-F | ATGGATTCCTATCTCAGTGGAAAAGGACATAATTATCCGGAAACT |
| Pnao-W220Y-N224K-R | ATTATGTCCTTTTCCACTGAGATAGGAATCCATCATATCCTTTTG |
| Pnao-W220Y-N224D-F | ATGGATTCCTATCTCAGTGGAGATGGACATAATTATCCGGAAACT |
| Pnao-W220Y-N224D-R | ATTATGTCCATCTCCACTGAGATAGGAATCCATCATATCCTTTTG |
| Pnao-W220Y-N224E-F | ATGGATTCCTATCTCAGTGGAGAGGGACATAATTATCCGGAAACT |
| Pnao-W220Y-N224E-R | ATTATGTCCCTCTCCACTGAGATAGGAATCCATCATATCCTTTTG |
| Pnao-W220Y-N224C-F | ATGGATTCCTATCTCAGTGGATGTGGACATAATTATCCGGAAACT |
| Pnao-W220Y-N224C-R | ATTATGTCCACATCCACTGAGATAGGAATCCATCATATCCTTTTG |
| Pnao-W220Y-N224W-F | ATGGATTCCTATCTCAGTGGATGGGGACATAATTATCCGGAAACT |
| Pnao-W220Y-N224W-R | ATTATGTCCACCTCCACTGAGATAGGAATCCATCATATCCTTTTG |
| Pnao-W220Y-N224R-F | ATGGATTCCTATCTCAGTGGACGTGGACATAATTATCCGGAAACT |
| Pnao-W220Y-N224R-R | ATTATGTCCACGTCCACTGAGATAGGAATCCATCATATCCTTTTG |
| Pnao-W220Y-N224G-F | ATGGATTCCTATCTCAGTGGAGGTGGACATAATTATCCGGAAACT |
| Pnao-W220Y-N224G-R | ATTATGTCCACCTCCACTGAGATAGGAATCCATCATATCCTTTTG |
| Pnao-W220Y-N224A-F | ATGGATTCCTATCTCAGTGGAGCTGGACATAATTATCCGGAAACT |
| Pnao-W220Y-N224A-R | ATTATGTCCAGCTCCACTGAGATAGGAATCCATCATATCCTTTTG |

The sequences that are homologous to pET-28a are in red. The codes with underline are mutation sites.
